# Integrated chromatin and transcriptomic profiling reveals sex-specific mechanisms of gene regulation in hepatic nutrient responses

**DOI:** 10.1371/journal.pbio.3003601

**Published:** 2026-02-12

**Authors:** Zhengyi Zhang, Vivien Su, Carrie B. Wiese, Lijing Cheng, Dan Wang, Ya Cui, Aneesh Kallapur, Jason Kim, Xiaohui Wu, Peter H. Tran, Zhenqi Zhou, David Casero, Wei Li, Andrea L. Hevener, Karen Reue, Tamer Sallam

**Affiliations:** 1 Division of Cardiology, Department of Medicine, University of California, Los Angeles, Los Angeles, California, United States of America; 2 Department of Physiology, University of California, Los Angeles, Los Angeles, California, United States of America; 3 Molecular Biology Institute, University of California, Los Angeles, Los Angeles, California, United States of America; 4 Human Genetics, David Geffen School of Medicine, University of California, Los Angeles, Los Angeles, California, United States of America; 5 Division of Computational Biomedicine, Biological Chemistry, University of California, Irvine, Irvine, California, United States of America; 6 Division of Endocrinology, Diabetes and Hypertension, Department of Medicine, University of California, Los Angeles, Los Angeles, California, United States of America; 7 F. Widjaja Inflammatory Bowel Disease Institute, Cedars-Sinai Medical Center, Los Angeles, California, United States of America; 8 Department of Medicine and VA Greater Los Angeles Healthcare System GRECC, Los Angeles, California, United States of America; 9 Iris Cantor-UCLA Women’s Health Research Center, Los Angeles, California, United States of America; Institutes of Biomedical Sciences, Shanghai Medical College, College of Life Science, Fudan University, CHINA

## Abstract

Little is known about how sex and diet interact at the level of chromatin organization. A comprehensive analysis of diet-induced chromatin dynamics can reveal how the liver mounts a rapid adaptive response to environmental cues and uncover mechanisms underlying sex differences. Here, we employed an integrative strategy to construct a nucleosome accessibility atlas of the mouse liver under different dietary conditions. Stringent analysis revealed a largely preserved hepatic chromatin landscape across feeding states, with sex being the critical factor driving changes in chromatin accessibility. Notably, lipid-rich diet preferentially enriched CCAAT-binding motifs in females, while nutrient-sensing nuclear receptor motifs were more strongly enriched in males. Furthermore, using the Four Core Genotypes model (XX ovaries / XY testes / XX testes / XY ovaries), we disentangled the effects of gonadal and chromosomal sex on diet-induced gene regulation. By leveraging this framework with multiple mouse models and molecular approaches, we identified a suppressive role of testosterone in regulating the sex-dimorphic GWAS gene *PNPLA3*. Overall, we establish an unbiased transcriptomic resource that revealed chromatin dynamics and identified gene clusters associated with distinct sex-related factors.

## Abstract 2

Metabolic diseases, including fatty liver diseases, are often influenced by biological sex. This study provides a detailed characterization of how sex and diet interact to shape molecular responses in the mouse liver, teasing apart how gonadal and chromosomal sex influence diet-induced transcriptomic and chromatin dynamics.

## Introduction

Epigenetic checkpoints help cells coordinate transcript biogenesis and crosstalk with other steps in gene regulation [1]. DNA is typically stored in nucleosomes, highly condensed structures that congregate to form chromatin [2]. Chromatin converts from an active to an inactive state through various mechanisms [3]. Chemical modifications of DNA or histone proteins can alter the three-dimensional structure of chromatin and position of nucleosomes [4,5]. Since nucleosomal rearrangements often precede binding and activation of transcription factors (TFs) [6,7], information about nucleosome position can be used to infer active regulatory regions. Genome-wide analysis of accessible chromatin regions has grown in popularity with the development of ATAC-seq [8,9]. Despite an increase in ATAC-seq datasets on the spatial organization of chromatin states in tissues under basal conditions or during development [10–12], our understanding of how chromatin rearrangements affect metabolic health and disease states are still ongoing and largely constrained to a few well-studied genes and broad epigenetic modifications [13–16]. In particular, few studies have carefully examined sex-related differences in nucleosome positioning in response to diet [17–19].

The liver transduces dietary signals to influence gene expression and metabolism [20,21]. For example, a high-fat diet (HFD) alters hepatic metabolism to accommodate the intake of excess calories and a high fat content, while a cholesterol-rich diet also alters hepatic metabolism and RNA biogenesis but does so differently than a HFD [22–25]. Both diets can increase the risk of metabolic-associated fatty liver disease (MAFLD) [26–28]. MAFLD and other metabolic traits are influenced by biological sex [29–31]. For example, hepatic lipid composition at baseline and after feeding a lipid-rich diet differs by sex [23,31]. Proposed mediators of sex differences in hepatic gene expression and metabolism include estrogen receptor, growth hormone, X-chromosome factors, and the BCL6–STAT5 axis [23,32].

Changes in chromatin can lay the foundation for transcription or interactions with pathways involved in transcript splicing, modification, and export [33–35]. However, we do not know the extent to which the hepatic chromatin environment changes in response to different challenges or how it contributes to gene expression and function. Since selective gene regulation is strongly influenced by epigenetics [36–38], mapping chromatin accessibility can be used to infer critical transcriptional regulators and determine their cooperative and hierarchical interactions. In this resource study, we mapped the chromatin landscape in mouse liver and analyzed the effects of diet and sex on hepatic transcriptional regulation.

## Results

### Sex regulates chromatin accessibility at promoter regions in liver

**Fig 1:**
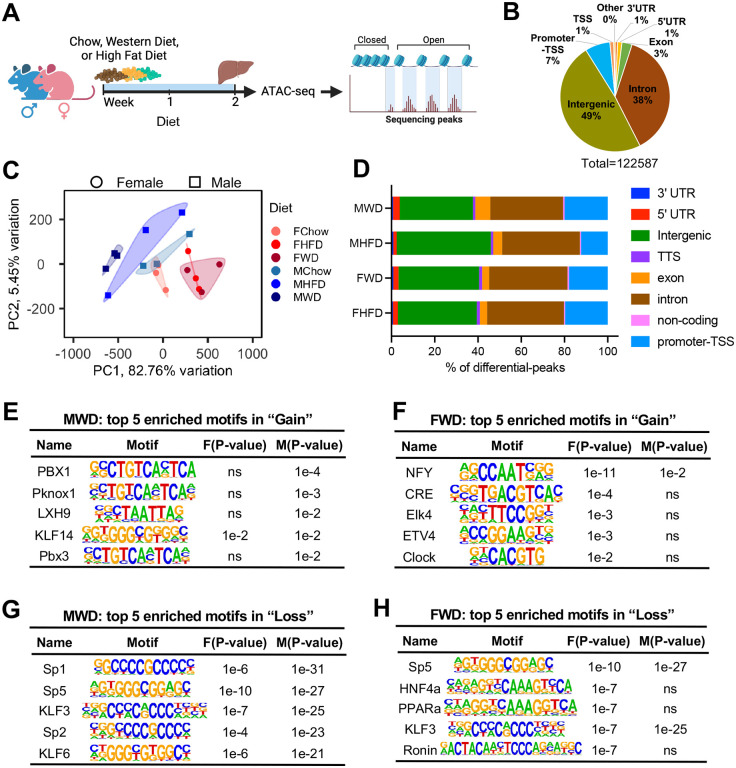
Sex regulates hepatic chromatin accessibility in response to diet. **(A)** Design of liver ATAC-seq experiments (*n* = 3 mice per group). **(B)** Distribution of genomic locations for all peaks. **(C)** PCA plots of hepatic chromatin accessibility generated from the peak score of each sample (*n* = 122,587). (FChow) females on CD, (FWD) females on WD, (FHFD) females on HFD, (MChow) males on CD, (MWD) males on WD, (MHFD) males on HFD. **(D)** Genomic location of peaks that showed gain or loss of accessibility in female or male mice liver. **(E)** Top five enriched motifs by HOMER using top 200 peaks from male WD/CD (ranked by fold changes, *P* < 0.05). **(F)** Top five enriched motifs by HOMER using top 200 peaks from female WD/CD (ranked by fold changes, *P* < 0.05). **(G)** Top five enriched motifs by HOMER using bottom 200 peaks from male WD/CD (ranked by fold changes, *P* < 0.05). **(H)** Top five enriched motifs by HOMER using bottom 200 peaks from female WD/CD (ranked by fold changes, *P* < 0.05).

To better understand how diet affects chromatin rearrangements, we fed male and female wildtype mice a normal chow diet (CD), a cholesterol-rich Western diet (WD), or a low-cholesterol HFD for 2 weeks and examined their livers by ATAC-seq (Fig 1A). Comparable read counts were obtained across all groups after sequencing, and each sample exhibited a similar alignment rate (S1A and S1B Fig). Of 122,587 unique peaks (indicating accessible chromatin) mapped in the three diet groups (Fig 1B, S1 Table), 49% were in intergenic regions, 38% were in introns, and 7% were in “promoter–transcription start site (TSS)” regions (Fig 1B). Principal component analysis showed that female and male mice fed a CD had largely similar profiles for principal components 1 and 2 (Fig 1C). Principal component 1 captured >82% of the variance (Fig 1C). Male mice responded more variably to diet than females, particularly when fed a HFD (Fig 1C). Changes in chromatin in response to WD or HFD differed by sex, and WD led to greater differences in principal component 1. Thus, hepatic chromatin accessibility in response to a lipid-rich diet differed in a highly sex-biased manner.

Most accessible sites did not show altered chromatin accessibility in response to diet (constitutive); however, females had both greater loss and gain of accessible sites than males (S1C Fig, S1 Table). The percentage of differentially-accessible regions (DARs) due to a WD or HFD was high in the intergenic, intronic, and promoter-TSS regions, which did not reveal notable sex-specific differences in genomic localization (Fig 1D, S1 Table). However, sex-induced differences in chromatin accessibility were proportionally more clustered in the TSS region (S1D–S1G Fig). Compared to CD, female mice fed a WD or HFD had more DARs, including both gain and loss of accessible sites at promoters, than males on the same diet (S1H and S1I Fig). Thus, chromatin rearrangements in response to a lipid-rich diet differed between male and female mice, particularly in the promoter regions.

To identify potential TFs involved in sex-specific chromatin regulation, we first analyzed motif enrichment, using the top 200 DARs from each genomic location in each group (CD versus HFD or WD). Given the lower individual variability in the WD group (Fig 1C), we selected the WD as the representative condition for subsequent analyses. Comparison of the top 5 enriched motifs in the WD group versus the CD group showed that male “gain” peaks were specifically enriched with PBX1 and Pknox1, whereas females’ “gain” peaks were enriched in TFs from with NFY, CRE, and ETS families (Elk4 and ETV4) (Fig 1E and 1F). Notably, NFY was enriched in both males and females, but the degree of enrichment varied by sex. For the “loss” peaks, males and females shared common top enriched motifs, including zinc-finger DNA-binding proteins from Sp TF family (Sp1, Sp2, and Sp5), and KLF3, with higher enrichment in male mice. The TFs distinctly enriched in female “loss” included PPARα and HNF4α (Fig 1G and 1H), two nuclear receptors whose activities are known to differ in males and females [39–41]. Thus, we observed sex bias in specific TF motif enrichment in response to WD feeding.

### Motif analysis reveals a sex-biased TF preference in response to diets

**Fig 2:**
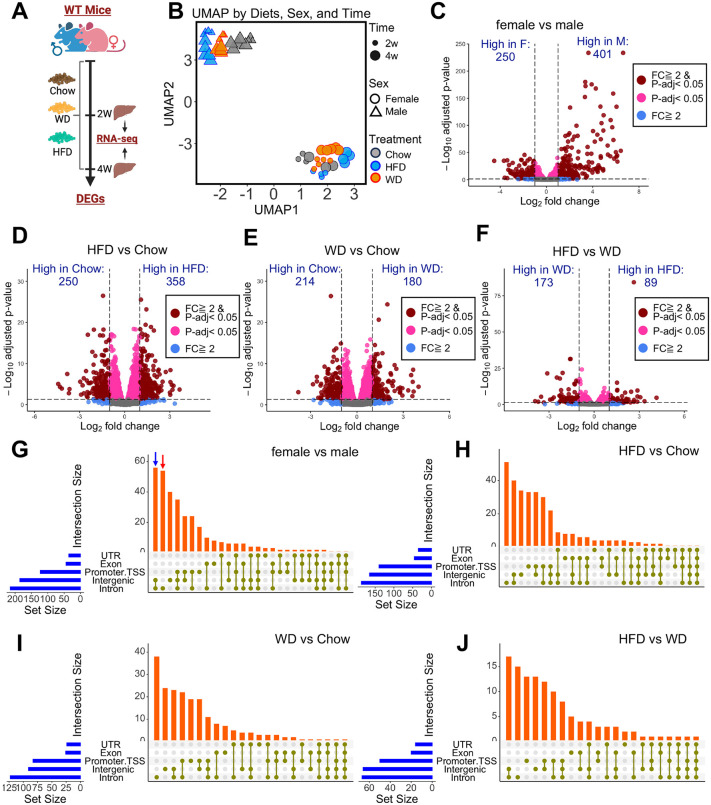
Relationship of DEGs to chromatin accessibility. **(A)** Schematic of RNA-seq analysis of mouse liver. **(B)** UMAP generated by using reads per kilobase per million mapped reads (RPKM) of RNA-seq. **(C–F)** Volcano plots showing DEGs as indicated. **(G–J)** UpSet plots showing the genomic distribution of ATAC-seq peaks associated with DEGs across five genomic regions. The “intersection size” represents the number of DEGs which showed ATAC-seq peaks within the gene overlapping genomic regions. For example, the blue arrow in Fig 2G reveals that approximately 55 DEGs have peaks located in both intronic and intergenic regions. The red arrow highlights DEGs with peaks exclusively in intronic regions. The “set size” denotes the total number of peaks identified in each genomic category. For example, more than 200 intronic peaks are associated with DEGs in Fig 2G.

To characterize the relationship between changes in hepatic chromatin accessibility and gene expression, we first analyzed the hepatic transcriptome by RNA-seq after 2 or 4 weeks on a WD, HFD, or CD (Fig 2A). Consistent with the ATAC-seq results, WD and HFD induced similar patterns of gene expression changes, but transcriptomic profiles were distinctly separated by both sex and diet. According to UMAP analysis, sex was the primary contributing factor (Fig 2B). Therefore, we first examined the global effects of sex and identified 401 genes with higher expression in males and 250 genes with higher expression in females (Fig 2C). We also assessed the global effects of diet by comparing HFD versus CD, WD versus CD, and HFD versus WD. Both the HFD and the WD induced a substantial number of differentially expressed genes (DEGs) comparing to CD (Fig 2D and 2E), but a relatively small number of DEGs were identified for HFD versus WD (Fig 2F), and as expected, these were related to sterol biosynthesis (S2A Fig). We next aimed to understand how changes in chromatin accessibility relate to changes in gene expression. Because a single DEG may be associated with multiple regions of accessible chromatin, we assigned ATAC-seq peaks to DEGs based on their genomic locations to determine which regions may contribute to the regulation of these genes. Peaks were predominantly mapped to intronic, intergenic, and promoter-TSS regions, and most DEGs showed peaks in more than one genomic location (Fig 2G–2J).

**Fig 3:**
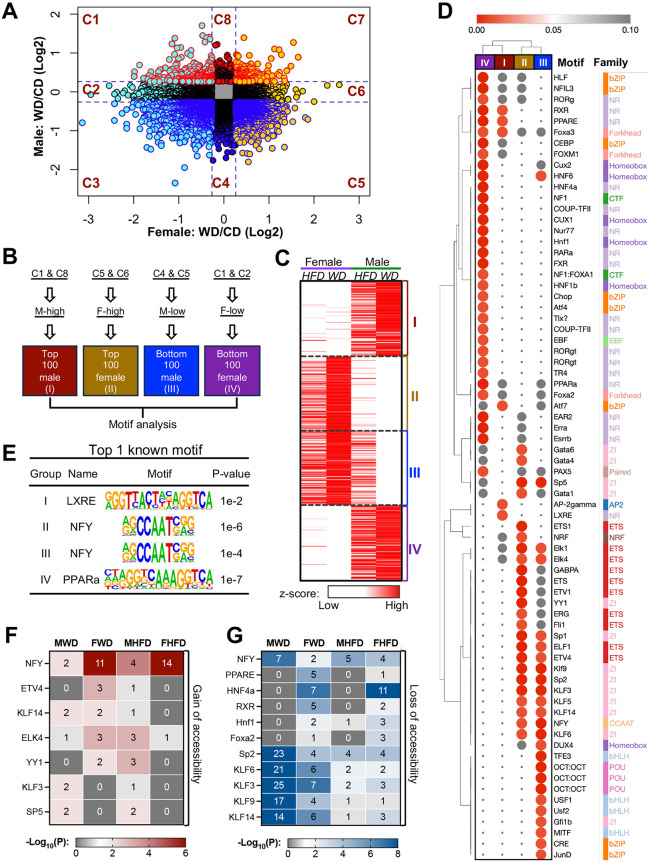
Diet induces sex-divergent motif enrichment in liver. **(A)** Correlation analysis of ATAC-seq peaks in male and female mice fed a WD. Dashed lines indicate 1.2-fold change. **(B)** Strategy for analysis of motifs by group. Roman numerals represent the peak groups selected for motif analysis, which were subsequently used in Fig 3D and 3E. **(C)** Heatmap showing the relative level of peaks selected from Fig 3B, separated by group. **(D)** Enrichment of motifs in different groups in Fig 3B and motif families identified with HOMER. Color in heatmap represents the *P* value. **(E)** Top single motif from each group from Fig 3D. **(F and G)** Motif analysis by HOMER using the top 200 peaks (Gain) or bottom 200 (Loss) promoter-specific peaks from each group. Numbers in heatmaps indicating the –Log_10_(P-value). MWD: male WD vs. CD; FWD: female WD vs. CD; MHFD: male HFD vs. CD; FHFD: female HFD vs. CD. All selected peaks differed from those in mice fed a CD (*P* < 0.05 calculated with DEseq2).

To further disentangle at a genome-wide scale the factors that led to sex-specific chromatin responses to hyperlipidemia, we analyzed male- and female-biased DARs in the WD and CD groups (Fig 3A). Four groups emerged: Groups I (males) and II (females) had gains in chromatin accessibility in response to a WD, whereas Groups III (males) and IV (females) had losses (Fig 3B). We chose the top and bottom 100 peaks from each group for motif analysis. These peaks also exhibited a consistent pattern in HFD versus CD (Fig 3C). The results of motif analysis revealed distinct sex-biased motif differences (Fig 3D). For example, Cux2 was specifically enriched in group IV (“female loss” of accessibility), consistent with reports that Cux2 enrichment in genomic regions with greater hepatic chromatin accessibility tends to be greater in male than in female mice [18]. Female-biased clusters also showed enrichment of HNF4α motif as expected, consistent with our earlier results (Fig 1F) and prior reports [39–41]. The peaks in the “male gain” (group I) and “female loss” (group IV) were enriched in motifs of nutrient-sensing nuclear receptor family members (e.g., LXRE, RXRE, and PPARE), whereas the peaks in the “female gain” (group II) and “male loss” (group III) were enriched in motifs of the zinc finger protein family, ETS family, and NFY (CCAAT) (Fig 3D). The top enriched motifs for gains in chromatin accessibility were LXRE in males and NFY in females (Fig 3E).

Since promoter regions contained the highest number of enriched motifs, we conducted promoter-specific motif analysis using the top 200 peaks with increased accessibility, and the bottom 200 peaks with decreased accessibility in HFD and WD groups as compared to the CD group, in both sexes (ranked by fold change (FC), with *P* value < 0.05). Interestingly, the major TFs identified were largely consistent with those from the motif analysis in Fig 3B, except for SP5, which showed opposite enrichment (Fig 3F and 3G). Notably, although a substantial number of non-promoter peaks were detected overall (Fig 1B), most peaks analyzed in Fig 3B were in promoter regions (S2B Fig). Furthermore, LXR motifs remained enriched in an analysis of the top 300 peaks, indicating chromatin accessibility in male mice fed a WD (S2C Fig, ranked by FC, with *P* value < 0.05), supporting the reliability of the motif analysis strategy used in Fig 3B. Together, these results suggest that, in response to different diets, males are more sensitive to chromatin rearrangements of nutrient-sensing TFs, whereas females are more sensitive to rearrangements of promoter-binding TFs such as NFY.

### Chromatin accessibility of sex-specific TFs are associated with distinct transcriptomic responses driven by gonadal or chromosomal factors

**Fig 4:**
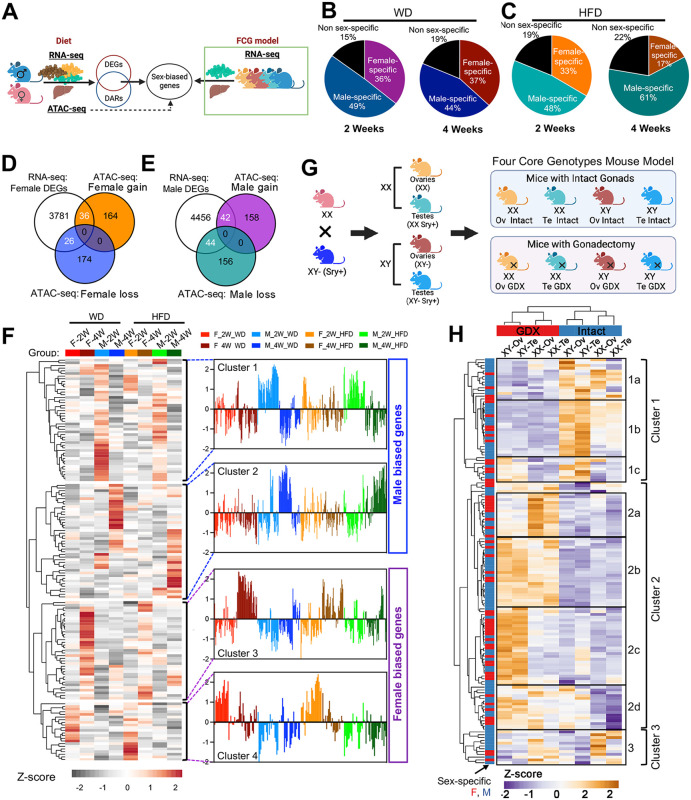
Gonadal and chromosomal factors influence sex-dimorphic DEGs. **(A)** Schematic of strategy of sex-biased gene identification. **(B and C)** DEGs (fold change >1.5, *P* < 0.05) in male or female mice fed a WD (B) or HFD (C) for 2 or 4 weeks. **(D and E)** Venn diagrams of DEGS and the top and bottom 200 DARs (from Fig 3A) in female (D) and male (E) mice. **(F)** Relative expression of overlapping genes identified in (D) and (E) based on RNA-seq from male (M) and female (F) mice after 2 (2W) or 4 (4W) weeks on a WD or HFD, shown relative to mice fed a CD at the corresponding time points. **(G)** Schematics of the strategy to generate the FCG mice used in the study *(left)* and of their genetic characteristics *(right)*. The mice were first divided into two main groups based on gonadal status: intact gonads (Intact) and gonadectomy (GDX). Within each category, further separation by sex chromosomes (XX or XY) resulted in a total of eight subgroups. Gene expression was then compared across these groups under each condition. **(H)** Expression of diet-induced, sex-biased genes (F) detected in FCG mice by RNA-seq. Clusters were generated based on counts per million reads (CPM). Ov, ovaries; Te, testes.

Differences in gene regulation between the sexes could be driven by changes in gonadal or chromosomal factors [32,42]. To better understand the contributions of these factors in dietary responses, we first analyzed hepatic DEGs identified by RNA-seq and overlapped them with DARs from ATAC-seq in a dietary challenge study. The resulting DEGs were further validated using transcriptomic data from the Four Core Genotypes (FCG) mouse model (Fig 4A). ATAC-seq results showed that WD or HFD induced or suppressed gene expression in similar patterns (S3A Fig). We first analyzed the expression of ~2000 genes differed in response to a WD or HFD versus a CD (S3B and S3C Fig, S2 Table). About 80% of these DEGs exhibited sex-biased expression, as judged from FCs and *P* values (Fig 4B and 4C, S2 Table), consistent with evidence that a high proportion of hepatic genes are regulated in a sex-biased manner [20]. We annotate the functions of DEGs in males and females in response to lipid-rich diets by Metascape [43] (S3D Fig). While the common DEGs induced by diets between sexes mapped to “metabolism of lipid” as expected (S3E Fig), male-specific DEGs and female-specific DEGs were enriched to different pathways (S3F–S3I Fig).

To explore the relationship between changes in chromatin accessibility and genes whose expression differed by sex, we overlapped DEGs from male or female RNA-seq with female or male ATAC-seq changes (Fig 4D and 4E). Clustering of gene expression by integrating ATAC-seq showed that the WD and HFD had similar overall effects on gene expression; however, gene expression in both the WD and HFD groups differed by the duration of the dietary challenge (Fig 4F). Furthermore, the gene expression patterns enabled us to predict the top-enriched motifs of male-biased and female-biased genes (S3J Fig). Among male-biased genes, the androgen receptor motif was strongly enriched in males but not in females. Conversely, among female-biased genes, the estrogen receptor (ESR1) motif was strongly enriched in females but not in males. These results align well with known hormonal regulation of hepatic gene expression in the two sexes. Unlike the ATAC-seq analysis, which showed distinct LXR enrichment in males (Fig 3D and 3E), the RNA-seq results showed LXR enrichment in both males and females (S3J Fig). Thus, the RNA analysis confirmed most but not all of the sex-biased results of chromatin dynamics.

The two primary determinants of biological sex are sex chromosomes (XX/XY) and gonads (ovaries/testes and the hormones they secrete) [44,45]. To further investigate their role in sex-biased gene expression in response to a lipid-rich diet, we used the FCG mouse model (Fig 4G), which has been widely used to reveal how sex-bias arises from distinct biological sources in metabolism and other physiological systems [32,42,46]. In this model, the *Sry* gene is relocated from the Y chromosome to an autosome, by which chromosomal sex (XX versus XY) from gonadal sex (testes versus ovaries) was effectively separated. The design of this model generates four genotypes: XXF, XYF, XXM, and XYM, allowing independent assessment of hormonal and chromosomal influences. This approach enabled us to determine whether hepatic transcriptional and chromatin responses to dietary challenges are primarily driven by gonadal hormones or by sex chromosome complement. We assessed the determinants of the expression of 148 sex-biased genes (Fig 4F) in FCG mice that had intact gonads or had their gonads removed (GDX) in adulthood and were fed a HFD. About 80% of these genes were also regulated by a sex determinant (gonadal sex, chromosomal sex, or both) in the FCG mice (S3K and S3L Fig, S3 Table).

Both gonadal and chromosomal status affected the expression of many sex-biased genes (Figs 4H, S3L Fig, and S3 Table). Cluster 1 included genes whose expression decreased after GDX, whereas Cluster 2 included genes whose expression increased after GDX (Fig 4H). Within these groups, many genes were influenced by chromosomal sex, with clear patterns of greater expression in XX than XY liver (e.g., Cluster 2a in GDX mice) or greater expression in XY than XX liver (e.g., Cluster 1b in intact mice and Cluster 2c in GDX mice). These findings reveal the complex regulation of gene regulatory mechanisms in response to diet, which was influenced by both genetic and hormonal sex determinants. In many cases, the regulation by sex chromosome genotype was more apparent after gonadal hormones were depleted by GDX, suggesting that sex chromosomes are an important determinant of diet responses in the setting of reduced gonadal hormone levels (e.g., after menopause and andropause). In summary, we found overlap in sex-biased signatures, suggesting that changes in chromatin dynamics under different dietary conditions are functionally contributing to changes in gene expression.

### Chromatin architecture informs mechanisms of sex differences in disease-relevant genes

**Fig 5:**
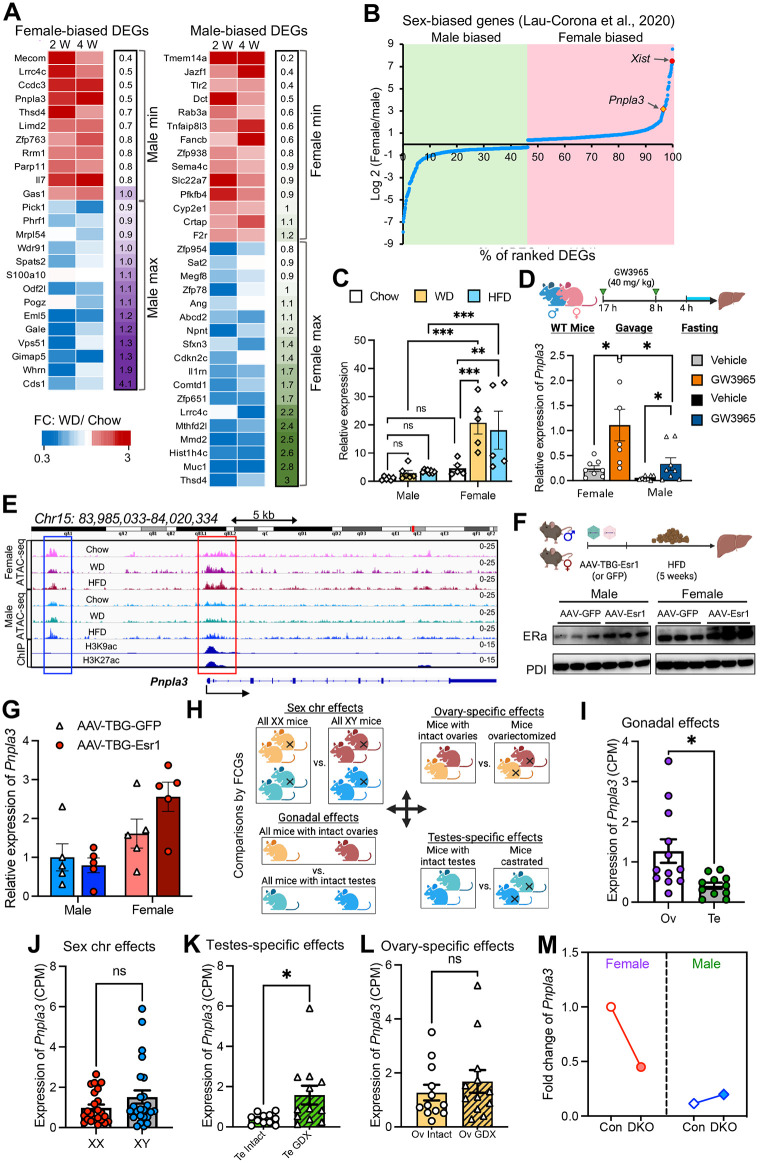
*Pnpla3* is regulated by male gonads and epigenetic factors. **(A)** Heatmap of fold changes (WD relative to CD) of sex-biased genes. *(left)* Female-biased DEGs. *(right)* Male-biased DEGs. **(B)** Sex-biased DEGs identified by RNA-seq. **(C)** *Pnpla3* expression determined by qPCR of livers of mice fed a WD (*n* = 6 males, 5 females), HFD (*n* = 8 males, 5 females), or CD (*n* = 6 males, 5 females) for 2 weeks. Values are mean ± SEM. **P < 0.01, ***P < 0.001 by two-way ANOVA. (ns) not significant. **(D)** Schematic of LXR agonist treatment *(top)* and hepatic expression of *Pnpla3* detected by qPCR *(bottom)*. *n* = 7–8. *P < 0.05 by unpaired *t* test. **(E)** ATAC-seq peaks from this study and ChIP-seq of histone mark from the ENCODE project (GEO numbers: GSM1000153 [H3K9ac] and GSM1000140 [H3K27ac]). ATAC-seq peaks from all three samples of each group were superimposed. **(F)** Schematic of ER-α (Esra) overexpression by AAV and immunoblotting for Esra in murine livers (*n* = 5). **(G)** Expression of *Pnpla3* detected by qPCR in the livers of mice from panel F (n = 5). Differences were not significant by *t* test or ANOVA. **(H)** Schema*t*ic comparing *Pnpla3* expression in the FCG mouse model. **(I)** Gonadal effects were assessed by comparing hepatic *Pnpla3* levels between mice with intact ovaries (Ov, *n* = 12) and those with intact testes (Te, *n* = 11), as shown in Fig 5H. *P < 0.05 by unpaired *t* test. **(J)** Sex chromosome effects were assessed by comparing hepa*t*ic *Pnpla3* levels between mice carrying XX (*n* = 23) or XY (*n* = 24) chromosomes, as shown in Fig 5H. *P* > 0.05 by unpaired *t* test. **(K)** Testis-specific effects were assessed by comparing hepa*t*ic *Pnpla3* levels between mice with intact testes (Te Intact, *n* = 11) and those that underwent GDX (Te GDX, *n* = 12), as shown in Fig 5H. *P < 0.05 by unpaired *t* test. **(L)** Ovary-specific effects were assessed by comparing hepatic *Pnpla3* levels between mice wi*t*h intact ovaries (Ov Intact, *n* = 12) and those that underwent GDX (Ov GDX, *n* = 12), as shown in Fig 5H. *P* > 0.05 by unpaired *t* test. **(M)** Fold change in fragments per kilobase of transcript per million mapped fragments (FPKM) of *Pnpla3* in male and female mice wi*t*h double-knockout (DKO) of Ezh1 and Ezh2. FPKM data are from Lau-Corona and colleagues, 2020 [13]. Values are relative to those in female controls (Con). The data underlying this Figure can be found in S1 Data.

To further investigate the relation between diet and sex-biased gene expression, we used stringent criteria (outlined in Materials and methods) to identify 25 female-biased DEGs and 32 male-biased DEGs (Fig 5A), many of which showed sex-biased expression in human liver (S4A–S4C Fig, S4 Table). Notably, the gene encoding Patatin-like phospholipase domain-containing protein 3 (*Pnpla3*) was one of the top female-biased DEGs in both mouse and human liver (Figs 5A, S4B, and S4D). Hepatic *PNPLA3* is of interest because variants at this gene are associated with risk of MAFLD [47–50]. Analysis of public RNA-seq datasets [13] also suggested that *Pnpla3* is a top-ranked female-biased DEG (Fig 5B). Quantitative PCR (qPCR) of liver samples from mice fed different diets confirmed the female-bias in *Pnpla3* expression (Fig 5C). Notably, 16 of the 25 female-biased DEGs overlapped with those from public RNA-seq datasets [13]; *Pnpla3* expression differed most between the sexes (S4E and S4F Fig). Additionally, chromatin accessibility from human liver samples detected by ATAC-seq [51] revealed a modest but consistent increase in promoter accessibility of *PNPLA3* in females (S4G Fig).

*Pnpla3* is impacted by LXR activity [52], and our analysis showed that LXR motifs were differentially enriched between sexes, but have little effect on the transcriptome. To determine whether LXR contributes to sex-based differences in hepatic *Pnpla3* expression, we used two different strategies. First, we assessed changes in canonical LXR target genes under different dietary conditions by RNA-seq. LXR target gene expression did not differ by sex except for *Scd1* in male mice fed a WD for 2 weeks (S4H Fig). Second, we orally administered the LXR agonist GW3965 to male and female mice and examined gene expression by qPCR. GW3965 induced LXR targets, but hepatic LXR gene regulation did not differ substantially between the sexes (S4I Fig). Collectively, these results suggest sex subtly affects some LXR targets but has no major global effect. Even with maximal LXR activation, however, *Pnpla3* was confirmed as a female-biased gene by qPCR (Fig 5D). Overall, our data suggest that LXR regulates *Pnpla3* but is not a critical factor in sex-differences in its expression.

Since *PNPLA3* underlies sex-based differences in MAFLD [53], and the higher hepatic expression of *PNPLA3* in female liver may be driven by the ER-α [54], we examined chromatin accessibility at the *Pnpla3* locus in male and female liver. We identified a cluster of accessible regions at the *Pnpla3* promoter region extending into the gene body (red, Fig 5E) and another upstream region (blue). The upstream region was near ER binding sites and showed minimal difference in regulation between the sexes. To directly examine the role of the ER-α in the sex bias in *Pnpla3* expression, we used an adeno-associated virus (AAV) under control of a TBG promoter to express ER-α (AAV-TBG-ESRa) or GFP (AAV-TBG-GFP) in wild-type male and female mice. Robust overexpression was confirmed by immunoblotting and qPCR, which also revealed increased expression of *Polg1*, an ER-α regulated gene we previously identified (Figs 5F, S5A, and S5B Fig) [55]. *Pnpla3* expression measured by qPCR showed no significant difference between AAV-GFP and AAV-ESRa in either males or females (Fig 5G). These results suggest that estrogen may not be the dominant factor in the observed sex-dependent differences in *Pnpla3* regulation.

Next, we assessed the effects of sex chromosomes, gonadal status, and ovaries/testes on *Pnpla3* expression in FCG mice (Fig 5H). Expression levels were higher in genotypes with ovaries than in those with testes (Fig 5I), and the XX chromosome and XY chromosome groups did not differ significantly (Fig 5J). Removal of testes increased *Pnpla3* expression (Fig 5K), whereas removal of ovaries had no effect (Fig 5L). These results suggest that *Pnpla3* expression was suppressed by testosterone rather than regulated by ovarian hormones.

Chromatin accessibility in the *Pnpla3* promoter region differed considerably between male and female mice fed different diets (Fig 5E, red). Notably, accessibility at the *Pnpla3* promoter was markedly reduced in male mice (Fig 5E). Analysis with PROMO [56,57], a method that constructs positional weight matrices from known TF binding sites for a given gene, supported the notion that potential hotspots around the promoter region of *Pnpla3* underlie the differences in expression under different conditions (S5C Fig). Interestingly, the promoter-accessible region overlapped with key histone marks that may control chromatin access (Fig 5E). Chromatin immunoprecipitation (ChIP) analysis of liver revealed stronger binding of H3K27ac at the *Pnpla3* locus in females than in males (S5D and S5E Fig), suggesting the chromatin stage could be relevant to *Pnpla3* sex-specific expression. This is partially supported by evidence that liver-specific double KO of Ezh1 and Ezh2, chromatin-modifying factors that orchestrate the balance of activating and repressive histone marks, attenuates the sex difference in *Pnpla3* expression (Fig 5M), consistent with previous links between Ezh1/2 and sex-biased regulation [13,47,58].

### Testosterone suppresses *Pnpla3* expression by inhibiting NFY chromatin interaction

**Fig 6:**
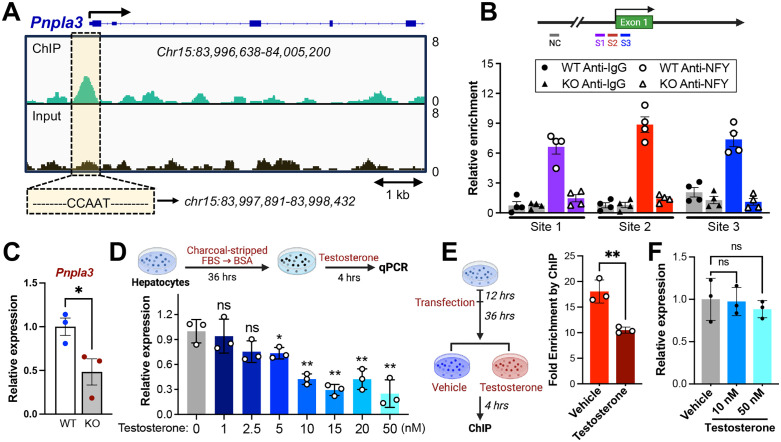
Testosterone suppresses *Pnpla3* through modulation of NFY. **(A)** Representative NFY ChIP-seq peaks at promoter region of *Pnpla3* from public datasets. GEO number: SRR1239519 (ChIP), SRR1239518 (input). **(B)** ChIP-qPCR performed in NYFA-WT or NFYA-KO primary hepatocytes treated with 5 μM GW3965 for 2 h (*n* = 4). **(C)** *Pnpla3* level detected by qRT-PCR from NFYA WT or KO primary hepatocytes treated with 5 μM GW3965 (5 μM) for 2 h (*n* = 3). **(D)** *Pnpla3* detected by qPCR from primary hepatocytes of WT mice, followed by treated with different concentrations of testosterone (*n* = 3). **(E)** ChIP-qPCR performed using primary hepatocytes with NFY overexpression and followed by testosterone (10 nM) or vehicle treatment for 4 h. Both groups were treated with GW3965 (5 μM) at the time adding vehicle or testosterone. Primers Site 2 from Fig 6B was used for detection (*n* = 3). **(F)** *Pnpla3* detected by qPCR from NFYA-KO primary hepatocytes treated with testosterone using same strategy as Fig 6D (*n* = 3). Data shown as mean ± SD. *: *P* < 0.05, **: *P* < 0.01, ns: *P* > 0.05. Unpaired *t* test performed for C and E; one-way ANOVA performed for D and F. The da*t*a underlying this Figure can be found in S1 Data.

NFY, a TF that interacts with chromatin to help maintain nucleosome-free regions at gene promoters [59], was among the top female-specific enriched TFs (Figs 1 and 3). Analysis of public ChIP datasets revealed a prominent NFY binding peak at the promoter of *Pnpla3* (Fig 6A). To investigate the potential regulation of NFY on *Pnpla3*, we generated NFY KO hepatocytes (S6A Fig). ChIP-qPCR confirmed binding at *Pnpla3* WT but not in NFY-KO hepatocytes (Fig 6B). Additionally, NFY KO led to a significant reduction in *Pnpla3* expression (Fig 6C). Since our FCG model demonstrated testosterone-mediated suppression of *Pnpla3*, we hypothesized that NFY chromatin interaction at *Pnpla3* promoter was regulated by testosterone. To test this, we first treated WT primary hepatocytes with testosterone and confirmed that testosterone reduced *Pnpla3* levels in a dose-dependent manner (Fig 6D). We next examined the effect of testosterone on NFY chromatin affinity, showing that testosterone treatment reduced NFY binding at the *Pnpla3* promoter (Figs 6E and S6B Fig). Importantly, testosterone treatment did not significantly alter *Pnpla3* expression in NFY-KO hepatocytes (Fig 6F), suggesting that testosterone’s inhibitory effect on *Pnpla3* is NFY dependent.

Collectively, we disentangle the contributions of distinct sex factors to diet-induced chromatin remodeling and gene expression, demonstrating that CCAAT-binding protein, in concert with testosterone signaling regulate promoter accessibility and contributes to sex-specific differences in *Pnpla3* expression.

## Discussion

This study provides comprehensive information on differences in chromatin dynamics between the sexes under different dietary conditions. We found that diet composition minimally impacted chromatin accessibility relative to the effect of sex. A lipid-rich diet led to greater chromatin accessibility at promoter regions with stronger enrichment of promoter-binding factors, such as the trimeric complex NFY, in female mice, and to stronger binding of nutrient-sensing nuclear receptors in male mice. Additionally, our findings provide insights into the regulation of the critical sex-dimorphic gene *Pnpla3* and reveal the complexity of murine hepatic chromatin dynamics in response to diet and sex.

Integrating chromatin dynamics and gene expression revealed substantial overlap in sex-biased signatures and provided evidence that nucleosome rearrangements influence gene expression changes. However, this was not the case for every enriched TF. For example, LXR-dependent chromatin differences between the sexes were pronounced, but differences in LXR-dependent gene expression were minimal. Although LXR binding patterns or activity might differ between the sexes, mechanisms beyond RNA biogenesis could regulate steady-state transcript levels. For example, differences in mRNA stability, export, or translation efficiency between the sexes could be important contributors [23].

Our unsupervised analysis of sex-biased genes highlighted new critical factors involved in lipid metabolism regulation and provided a framework for investigators to use this resource to decipher the mode of regulation of genes of interest. In genome-wide association studies, *PNPLA3* is one of the few genes consistently implicated in MAFLD [47–50]. Epidemiologic and molecular studies suggest that *PNPLA3* regulation by estrogen contributes to the rise in MAFLD in older women [54], despite the decrease in estrogen levels during menopause. Examination of chromatin dynamics at the *Pnpla3* locus confirmed that changes at promoters appear to underlie differences between sexes. However, we found that sex differences in *Pnpla3* transcript levels in mice are not regulated by estrogen but by male gonadal factors, histone modifiers, and TFs acting at promoters. Our findings do not exclude a role for estrogen signaling in *Pnpla3* regulation but rather suggest that multiple regulatory factors are at play. We point out that the AAV-mediated ESR overexpression may not fully replicate the physiological actions of estrogen in vivo, particularly accounting for the dynamic changes in estrogen, hormonal fluctuations, species-specific differences, or interactions with other endocrine pathways. Thus, the molecular pathways regulating sex-based differences in human *PNPLA3* expression will continue to be the subject of future studies.

Our study has a number of limitations. Although sex was a pivotal driver of chromatin responses, the duration of the dietary perturbations was only 2 weeks. We picked this time point since prior studies suggest that gene expression changes in response to diet are more pronounced within the first 2 weeks and compensatory pathways can ensue despite a more pronounced metabolic disease state [60,61]. Second, hepatic gene expression and hormone responsiveness are known to change with age [62–64], which may limit the generalizability of our findings. For instance, the decline in testosterone levels observed in aged males could alter chromatin accessibility and gene regulation compared with younger mice. Therefore, the mechanisms identified in this study may be age-specific, and future investigations using aged models will be essential to determine whether these regulatory patterns are preserved over time.

## Materials and methods

### Mice and diet

All mouse experiments were approved by the UCLA Animal Research Committee and adhered to the guidelines in the *Guide for the Care and Use of Laboratory Animals* (National Institutes of Health) under protocol number: ARC-2006-161 (FCG mouse experiments) and ARC-2018-044-AM-01 (other mouse experiments). The mice were on the C57BL/6 background and housed under pathogen-free conditions in a temperature-controlled room at 22 °C with 50%–65% humidity and a 12-h light/dark cycle. Wildtype mice (strain 000664, The Jackson Laboratory) were fed a standard CD (Rodent Diet 20, 5,053, PicoLab), a WD (D12079B, Research Diets), or a HFD (D12492, Research Diets) ad libitum for 2 weeks before ATAC-seq and for 2 or 4 weeks before RNA-seq. The number of mice in each group for RNA-seq is provided in Table 1. FCG mice on a C57BL/6 congenic background from a UCLA-maintained colony were used as described [42,65]. In these mice, the testis-determining gene *Sry* on the Y chromosome was deleted, and an *Sry* transgene was inserted into an autosomal chromosome, which allows for independent segregation of gonad development and sex chromosome complement [66].

**Table 1:** Numbers of mice in each RNA-seq group with diets challenging.

Diet	No. of mice on diet	
	2 weeks	4 weeks
Chow	3 M, 3 F	5 M, 3 F
Western	3 M, 5 F	4 M, 5 F
High-fat	4 M, 4 F	5 M, 4 F

FCG mice underwent GDX at 10–11 weeks of age under isoflurane anesthesia as described [67]. FCG mice were fed the HFD ad libitum for 10 weeks (intact gonad cohort) or 16 weeks (GDX cohort). Before sacrifice, mice were fasted for 6 h, unless specified otherwise (e.g., in the LXR agonist treatment experiment). Livers were harvested from three mice in each group, flash-frozen in liquid nitrogen, and stored at –80 °C. In the ATAC-seq studies, three mice were used in each group. The number of FCG mice used for sequencing are specified in figure legends.

### AAV transduction

For AAV-mediated overexpression of Esr1, 8-week-old male or female C57BL/6J wildtype mice (*n* = 5 per group) received injections of AAV8.TBG.GFP or AAV8.TBG.ESRa (VectorBiolabs) (1 × 10^11^ genome copies per mouse) in the lateral tail vein. One week later, the mice were placed on the HFD for 5 weeks. Mice were sacrificed after a 6-h fast.

### LXR agonist experiments

The protocol for the LXR agonist experiments in mice is described elsewhere [61,68]. In brief, 9-week-old mice (*n* = 8 males and 7 females) were given GW3965 (Sigma), 40 mg/kg, by oral gavage 17 h and 8 h before sacrifice after a 4-h fast. GW3965 was dissolved in dimethyl sulfoxide (Sigma) and delivered in canola oil (Sigma); vehicle controls (*n* = 8 males and 7 females) received dimethyl sulfoxide and canola oil.

Total RNA was extracted with TRIzol (Invitrogen) and reverse transcribed with a homemade reverse transcriptase as described [69]. cDNA was quantified with real-time PCR and SYBR Green Master Mix (Diagenode) on a BioRad Real-Time PCR instrument. The primers for amplifying *Pnpla3* were sourced from a previous publication [70]. Primers for amplifying *Polg1* were from our previous publication [55]. Gene expression was normalized to the housekeeping gene *36B4* or as indicated in the figure legend.

### Immunoblot analysis

Proteins were extracted with RIPA lysis buffer (Boston Bioproducts) supplemented with a complete protease inhibitor cocktail (Roche). Protein samples were resolved on a 4%–12% NuPAGE Bis-Tris Gel (Invitrogen), transferred to a Hybond ECL membrane (GE Healthcare), and probed with primary antibodies against PDI (3501, Cell Signaling Technology; 1:1000), ER-α (06-935, Sigma-Aldrich; 1:1000), and NFYA (sc-17753X, Santa Cruz Biotechnology; 1:800).

### Primary hepatocytes isolation and cell culture

Mouse primary hepatocytes were isolated as we previously described with minor modifications [71]. Briefly, mice were anesthetized with ketamine/xylazine, and perfusion was performed through cannula with 50 mL of Hank’s solution 10 mL per minute, followed by 50 mL of Liberase solution (final concentration: 30 µg/mL; perfusion speed: 5 mL per minute). After perfusion, livers were gently dissociated using forceps, and the resulting cell suspension was filtered through a 70 µm cell strainer. Cells were then centrifuged at 50*g* for 3 min at 4 °C. The cell pellet was washed twice, resuspended, and plated in 6-well plates or 15-cm culture dishes in pre-warmed washing/plating medium (William’s Medium E supplemented with GlutaMAX, HEPES, Penicillin/Streptomycin, 1 µM dexamethasone, 100 nM insulin, and 5% charcoal-stripped fetal bovine serum). After 3 hourso of incubation, the medium was replaced with maintenance medium (William’s Medium E containing 1 µM dexamethasone, 100 nM insulin, and 0.2% BSA).

### Gene overexpression and gene deletion in vitro

For gene overexpression experiments, primary hepatocytes were transfected with a pcDNA3.1 plasmid expressing full-length NFYA cDNA (constructed by VectorBuilder). Transfection was performed 12 h after primary hepatocytes plating, using a ratio of 3 µg plasmid per million cells with Lipofectamine 3,000 Transfection Reagent (Thermo Fisher Scientific) according to the manufacturer’s instructions. To generate NFYA KO cells, primary hepatocytes from NFYA^flox/flox^ [72] were treated with adeno-associated virus serotype 8 (AAV8) under control of the TBG promoter (purchased from Penn Vector Core), using either AAV8.TBG.Cre (KO) or AAV8.TBG.GFP (WT control). AAV was added to the culture medium at a dose of 2 × 10¹⁰ genome copies per million cells. Overexpression from transfection experiments or KO efficiency from AAV transduction experiments were confirmed by immunoblotting.

### Testosterone treatment

For testosterone treatment experiments, charcoal-stripped fetal bovine serum (Cat#: A3382101, Thermo Fisher Scientific) was used in place of regular fetal bovine serum during primary hepatocyte isolation and culture for hormone depletion as described in our previous publication with minor modifications [73]. Following hormone depletion culture, cells were treated with testosterone (Cat#: T-037, Cerilliant) for 4 h with the concentration indicated in the figure legend.

### Chromatin Immunoprecipitation (ChIP)

ChIP followed by quantitative PCR (ChIP qPCR) experiments were performed as previously described with a few changes [71]. Briefly, 40 million primary hepatocytes for each replicate were crosslinked with 2 mM Disuccinimidyl Glutarate (DSG, Cat#: 20,593, Thermo Fisher Scientific) for 1 h, followed by 1% formaldehyde for 12 min (four replicates for each group). For ChIP using testosterone-treated cells, 20 million NFYA overexpressed cells for each replicate were crosslinked with 2 mM DSG for 1 h, followed by 1% formaldehyde for 12 min (three replicates for each group). Sonication was performed using a M220 Focused-ultrasonicator (Covaris) according to the manufacturer’s protocol using the setting parameter: Peak Power = 140, Duty Factor = 5.0, Cycles/Burst = 200, Duration = 10 min. Chromatin was immunoprecipitated with 6 μg antibodies against NFYA (Cat#: sc-17753X, Santa Cruz Biotechnology) overnight at 4 °C. The ChIP samples were analyzed by qPCR using primers listed in S5 Table. All values obtained were normalized to a negative control region.

### ATAC-seq and ATAC-seq analysis

Frozen liver samples were analyzed by ATAC-seq according to our optimized protocol [71]. Each sample was stained with trypan Blue (Thermo Fisher), and nuclei were counted with a hemocytometer. For each reaction, 75,000 nuclei were extracted and centrifuged at 500*g* for 5 min at 4 °C. ATAC-seq libraries were constructed with Nextera Tn5 enzyme and a DNA library preparation kit (Illumina) as described [9]. AMPure XP magnetic beads were used for size selection. Libraries were sequenced with a NovaSeq 6000 (read length: 2 × 100 bp) at the Broad Stem Cell Research Center Sequencing Core at UCLA.

Sequenced reads from each sample were individually aligned to the mouse genome (mm9, NCBIv37) with Bowtie2 [74]. Duplicate reads and reads mapped to the mitochondrial genome or aligned to unmapped contiguous regions were removed. MACS2 [75] using the option –nomodel –keep-dup all -q 0.01 –llocal 10,000 was used to call for accessible sites in each sample. DESeq2 (Version: 1.42.1) was used to identify differential peaks with a FC ≥ 2 and *P* value < 0.05 in line with prior studies [76,77]. To identify DARs influenced by individual factors, including diet and sex, we analyzed ATAC-seq peaks to test the effects of diet (comparing WD versus chow, HFD versus chow, and HFD versus WD) and sex (comparing females versus males). Reproducible peak sites in each sample were merged, and RPKM values were obtained with SeqMonk (Babraham Bioinformatics). Bedgraph files were generated with HOMER [78] and transformed to Integrative Genomics Viewer (IGV) tiled data file (TDF) file (.tdf) and visualized with IGV tools [79]. Genes were annotated with the annotation function in HOMER using the genome location of peaks. Promoter-specific and global motif enrichment was analyzed with HOMER Motif Analysis [78,80]. R package PCAtools [81] was used for principal component analysis (Fig 1C). The R package umap (version 0.2.10.0) was used for UMAP analysis. Peaks and their annotations are listed in supplemental S1 Table.

### RNA-seq and RNA-seq analysis

RNA-seq was done as described [23]. Briefly, total RNA was extracted with TRIzol (Invitrogen) and purified with the Qiagen RNeasy Mini Kit (Qiagen). RNA concentration was measured with a Nanodrop, and sequencing was done with a Hiseq3000 (read length: 1 × 50 bp) at the UCLA Technology Center for Genomics and Bioinformatics. RNA-seq analysis was done as described [23]. Each sample was processed independently, and RPKM values were calculated for each sample individually. DEGs in diet experiments were defined as follows: for upregulated genes, FC [(WD or HFD)/CD] > 1.5 and *P* < 0.05; for downregulated genes, FC [CD/(WD or HFD)] > 1.5 and *P* < 0.05. Statistics on DEGs are shown in S2 Table. Gene ontologies and transcription were analyzed with EnrichR [82–84] or Metascape [43] as described in the results or figure legends. Venn diagrams were generated with Venny [85] or the Venn diagram tool from Bioinformatics and Evolutionary Genomics (https://bioinformatics.psb.ugent.be/webtools/Venn/). Heatmaps were generated with Morpheus (https://software.broadinstitute.org/morpheus) or ClustVis [86]. Liver RNA-seq data were from a public dataset [13].

For Fig 5, gene expression in controls was calculated as female RPKM/male RPKM. DEGs were considered female-biased if the FC of WD/CD was >1.2 at both 2 and 4 weeks in females and ≤1 at either time point in males or if the FC in CD/WD was >1.2 at both 2 and 4 weeks in females and ≤1 at least at either time point in males. DEGs were considered male-biased if the FC of WD/CD was >1.2 at both 2 and 4 weeks in males and ≤1 at either time point in females or if the FC of CD/WD was >1.2 at both 2 and 4 weeks in males and ≤1 at either time point in females.

For the RNA-seq analysis of FCG mice, reads were aligned to the GRCm39 mouse genome (Genome Reference Consortium), and read counts per gene were quantified with STAR (version 2.7.10a). DEGs were analyzed with DESeq2 (version 1.36); contrast tests with reduced models were done to evaluate the gonadal effect (mice with intact ovaries versus mice with intact testes), chromosomal effect (mice with XX chromosomes versus mice with XY chromosomes), ovariectomy effect (ovariectomized mice versus mice with intact ovaries), and castration effect (castrated mice versus mice with intact testes).

### ChIP-seq analysis

ChIP-seq data from public datasets are listed in the figure legends. We reanalyzed the ChIP-seq data using downloaded fastQ files. Reads were aligned to mouse genome mm9 (NCBIv37/mm9) with Bowtie2 [74]. MACS2 [75] was used for peak calling. To visualize the peaks, HOMER was used to generate bedgraph files, which were transformed to TDF files and visualized with IGV tools [79,80]. Gene annotation was done with SeqMonk (“mRNA” setting; Babraham Bioinformatics).

## Supporting information

S1 Table ACTC-seq peaks and annotation. (XLSX)

S2 Table Statistic of RNA-seq DEGs. (XLSX)

S3 Table Expression of overlapped genes in FCG model. (XLSX)

S4 Table Sex-biased genes in Fig 5A and their expression in human. (XLSX)

S5 Table Primers and Primer sequences for ChIP-qPCR. (XLSX)

S1 Data The data underlying figures. (XLSX)

S1 Raw Images WB uncropped images. (PDF)

S1 Fig ATAC-seq in mouse liver (related to Fig 1). (**A**) Total read count of each ATAC-seq sample. Values are shown as log_10_(total read count). (**B**) Total alignment rate from each sample, calculated with bowtie2 using mm9 as the reference genome. (**C**) Proportion of total peaks that showed gain, loss, or no change in chromatin accessibility in male (M) and female (F) mice fed a WD or HFD relative mice fed a CD. Gain or loss of accessibility was defined as a fold change (FC) >2 (gain) and *P* < 0.05, or FC < 0.5 and *P* < 0.05 (loss). (**D** and **E**) ATAC-seq signal intensity of peaks in mice fed a WD relative to that in mice fed a CD. (**F** and **G**) ATAC-seq signal intensity of peaks in mice fed a HFD relative to that in mice fed a CD. (**H**) Number of peaks in the promoter–TSS region that differed in male and female mice, according to HOMER annotation. (**I**) Number of peaks in the promoter–TSS region that differed in male and female mice, according to HOMER annotation.
(PDF)

S2 Fig Pathway and motif analysis of accessible chromatin in liver (related to Figs 2 and 3). (**A**) GO annotation (Biological Process) was done using the DEGs from Fig 2F. (**B**) Distribution of genomic locations for selected peaks in Fig 3B. (**C**) CHEA3 motif prediction using top 300 global peaks (WD versus CD) in male mice (*P* < 0.05).
(PDF)

S3 Fig Sex-specific hepatic gene expression profiles exhibit different motif preferences (related to Fig 4). (**A**) Heatmap of data for all the genes detected by RNA-seq in mouse liver samples. (**B**) DEGs in male or female mice fed a WD for 2 or 4 weeks. (**C**) DEGs in male or female mice fed a HFD for 2 or 4 weeks. (**D**) Venn diagram showing common DEGS in females and males and sex-specific DEGs. (**E**) Metascape analysis of male and female common DEGs. The top 5 terms are shown. (**F–I**) Top 10 Metascape terms for female- or male-specific upregulated or downregulated genes. (**J**) Motifs enrichment prediction of male-biased or female-biased genes in Fig 4F. (**K**) Genes from Fig 4F matched to DEGs identified by RNA-seq in the FCG mouse model. *P* < 0.05 was considered significant. (**L**) Venn diagram of diet-induced sex-difference genes from Fig 4F and DEGs identified by RNA-seq in the FCG mouse model.
(PDF)

S4 Fig Gene expression data from human liver corroborates analysis of murine studies (related to Fig 5). (**A**) Heatmap of transcripts per million reads (TPM) of genes (listed in Fig 5A) in human liver. TPMs from Adult Genotype Tissue Expression (GTEx) Project. Genes from Fig 5A that had no corresponding human genes or were not express were removed. (**B** and **C**) Venn diagram of diet-induced DEGs (Fig 5A) and female-biased (B) and male-biased (C) GTEx genes. (**D**) Expression of human *PNPLA3* in top 10 tissues. Data from GTEx. (**E**) Venn diagram of DEGs (Fig 4F) and sex-biased hepatic genes. (**F**) Fold change (male/female relative to controls) of sex-biased DEGs in S4E Fig determined with data from Lau-Corona and colleagues, 2020. (**G**) Chromatin accessibility by ATAC-seq from human liver samples (GEO accession: GSE164870). All samples from study were included except samples with IDs 767 (GSM5021293), 485 (GSM5021297), 793 (GSM5021300), and 797 (GSM5021301) due to globally reduced peak signals observed in these datasets. (**H**) LXR target gene expression based on RNA-seq. (CF2 and WF2): female mice fed a CD or WD for 2 weeks, (CF4 and WF4), females fed a CD or WD for 4 weeks, (CM2 and CM4) males fed a CD for 2 or 4 weeks, (WM2 and WM4) males fed a WD for 2 or 4 weeks. Number of samples for each group are listed in Table 1. (**I**) Expression of LXR target genes detected by qPCR from liver. *n* = 8 per group. *P < 0.05, **P < 0.01, ***P < 0.001 by unpaired *t* test. (ns) not significant. The data underlying this Figure can be found in S1 Data.
(PDF)

S5 Fig Chromatin architecture and regulation of *Pnpla3* (related to Fig 5). (**A**) Hepatic expression of *Esr1* of mice from Fig 5F, determined by qPCR. *n* = 5. ***: *P* < 0.001 by unpaired *t* test. (**B**) Hepatic expression of *Polg1* of mice from Fig 5F, determined by qPCR. *n* = 5. *: *P* < 0.05 by unpaired *t* test. (**C**) PROMO-predicted TF binding sites around the TSS of *Pnpla3*, as indicated in the red box in Fig 5E. “Level” is the density of binding sites of TFs; a higher level indicates greater TF binding. (**D**) H3K27ac ChIP-seq peaks from public datasets with the following GEO ID numbers: SRR6756579 (female Rep 1), SRR6756580 (female Rep 2), SRR6756581 (female Rep 3), SRR6756582 (female Rep 4), SRR6756571 (male Rep 1), SRR6756572 (male Rep2), SRR6756573 (male Rep3), and SRR6756574 (male Rep 4). (**E**) Fold change calculated based on ChIP-seq enrichment in acetylation of lysine 27 of the histone H3 protein (H3K27ac) in male or female liver (Lau-Corona and colleagues, 2020). SeqMonk was used for gene annotation (mRNA). The data underlying this Figure can be found in S1 Data.
(PDF)

S6 Fig Efficiency of CCAAT-binding protein gene deletion or overexpression (related to Fig 6). (**A**) NFYA protein levels detected by immunoblotting. Protein was isolated 48 h post AAV infection (Materials and methods for details). Twenty micrograms of total protein were loaded per lane. (**B**) NFYA overexpression efficiency detected by immunoblotting. Primary hepatocytes from WT mice were transfected with empty vector (EV) or NFYA overexpression plasmid (OE). Protein was isolated 36 h post-transfection, with 15 µg total protein loaded per lane. These cells were further used for ChIP in Fig 6E.
(PDF)

## Abbreviations

AAV: adeno-associated virus
CD: chow diet
ChIP: Chromatin immunoprecipitation
DAR: differentially-accessible region
DEG: differentially expressed gene
FC: fold change
FCG: Four Core Genotypes
HFD: high-fat diet
IGV: Integrative Genomics Viewer
MAFLD: metabolic-associated fatty liver disease
*Pnpla3*: Patatin-like phospholipase domain-containing protein 3
qPCR: Quantitative PCR
TDF: tiled data file
TF: transcription factor
TSS: transcription start site
WD: Western diet
