# Supplementary material for: Integrated chromatin and transcriptomic profiling reveals sex-specific mechanisms of gene regulation in hepatic nutrient responses
Source: PLoS Biol. 2026 Feb 12;24(2):e3003601. doi: 10.1371/journal.pbio.3003601 (PMC12900309; doi:10.1371/journal.pbio.3003601)
Supplement: S1 Raw Images — (PDF) [file pbio.3003601.s007.pdf]

Fig. 5F

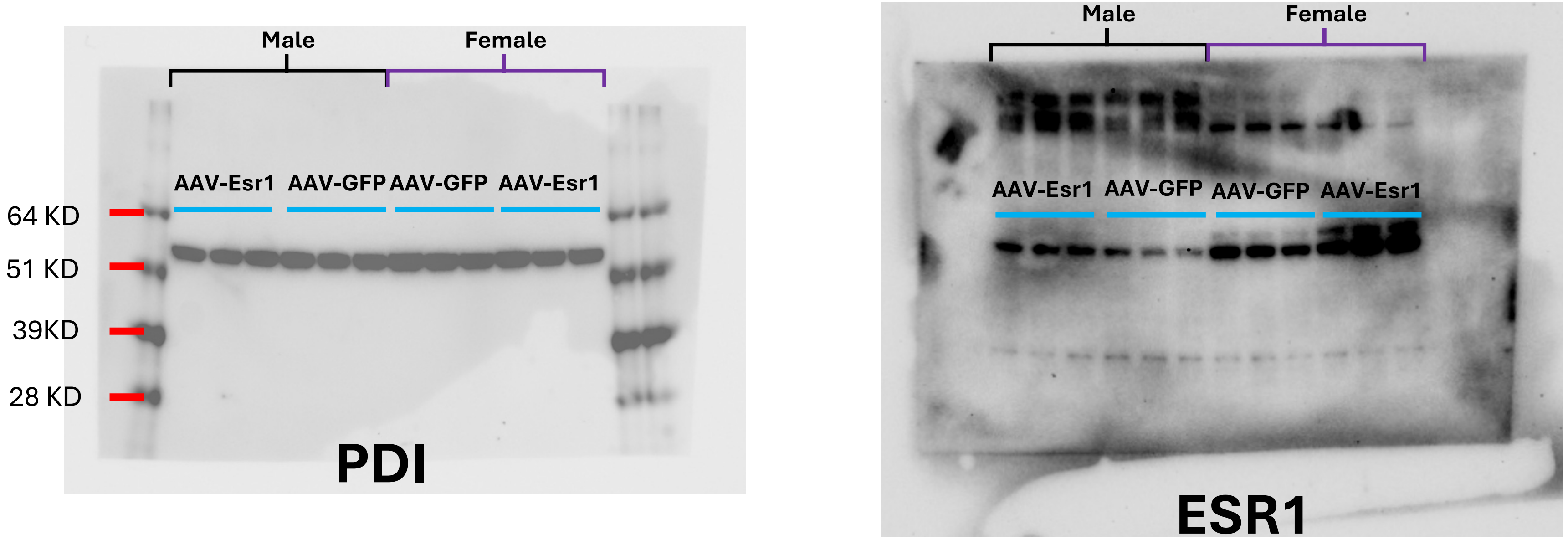

Protein Ladder: SeeBlue™ Plus2 Pre-stained
Protein Standard (Invitrogen)

S6A Fig.

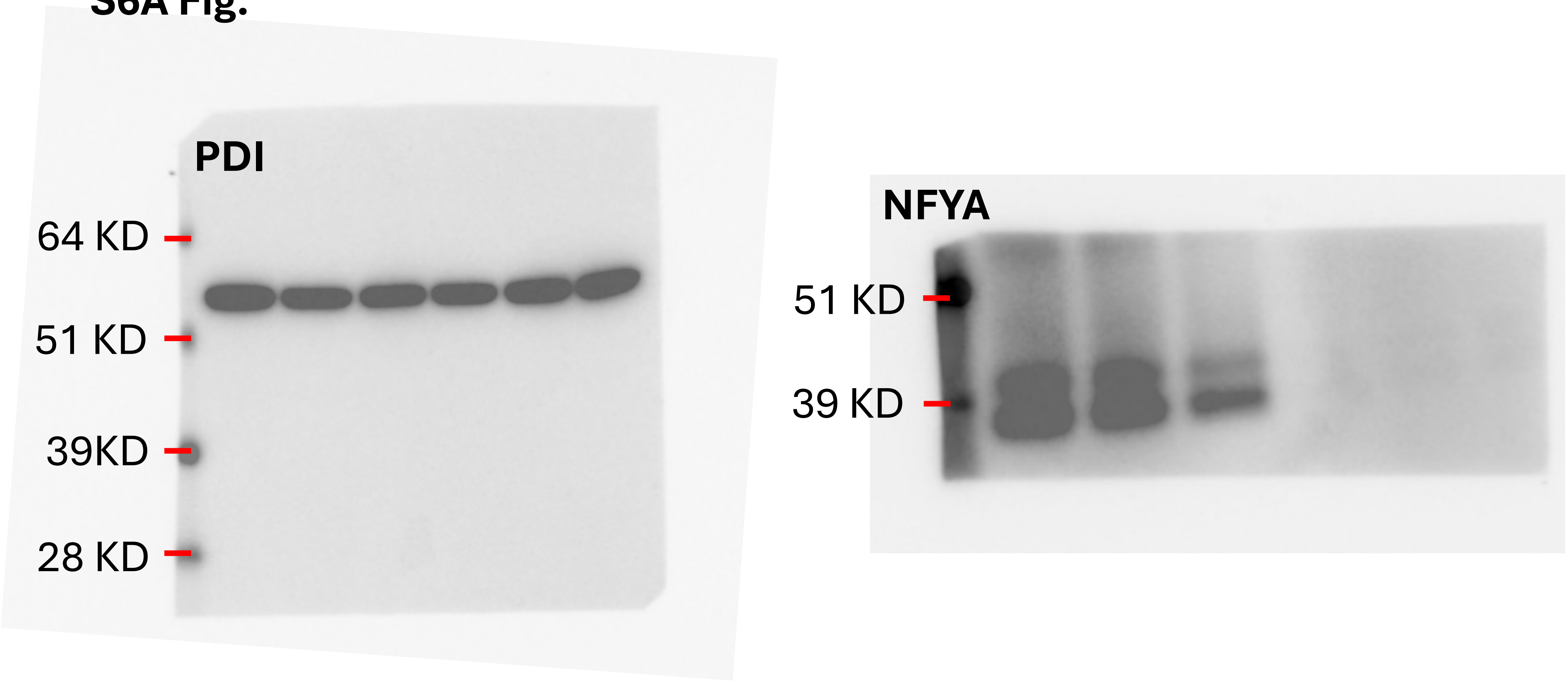

Protein Ladder: SeeBlue™ Plus2 Pre-stained Protein Standard (Invitrogen)

S6B Fig

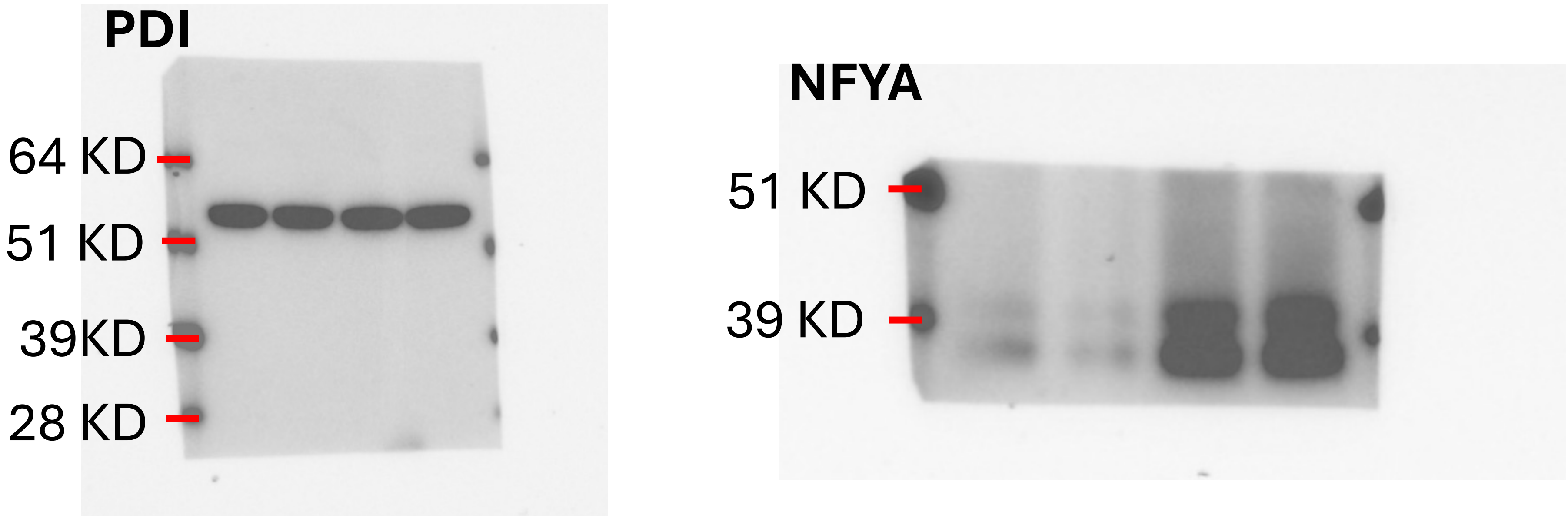

Protein Ladder: SeeBlue™ Plus2 Pre-stained Protein Standard (Invitrogen)
