# Supplementary figures and images for: Integrated chromatin and transcriptomic profiling reveals sex-specific mechanisms of gene regulation in hepatic nutrient responses
Source: PLoS Biol. 2026 Feb 12;24(2):e3003601. doi: 10.1371/journal.pbio.3003601 (PMC12900309; doi:10.1371/journal.pbio.3003601)

S1 Fig

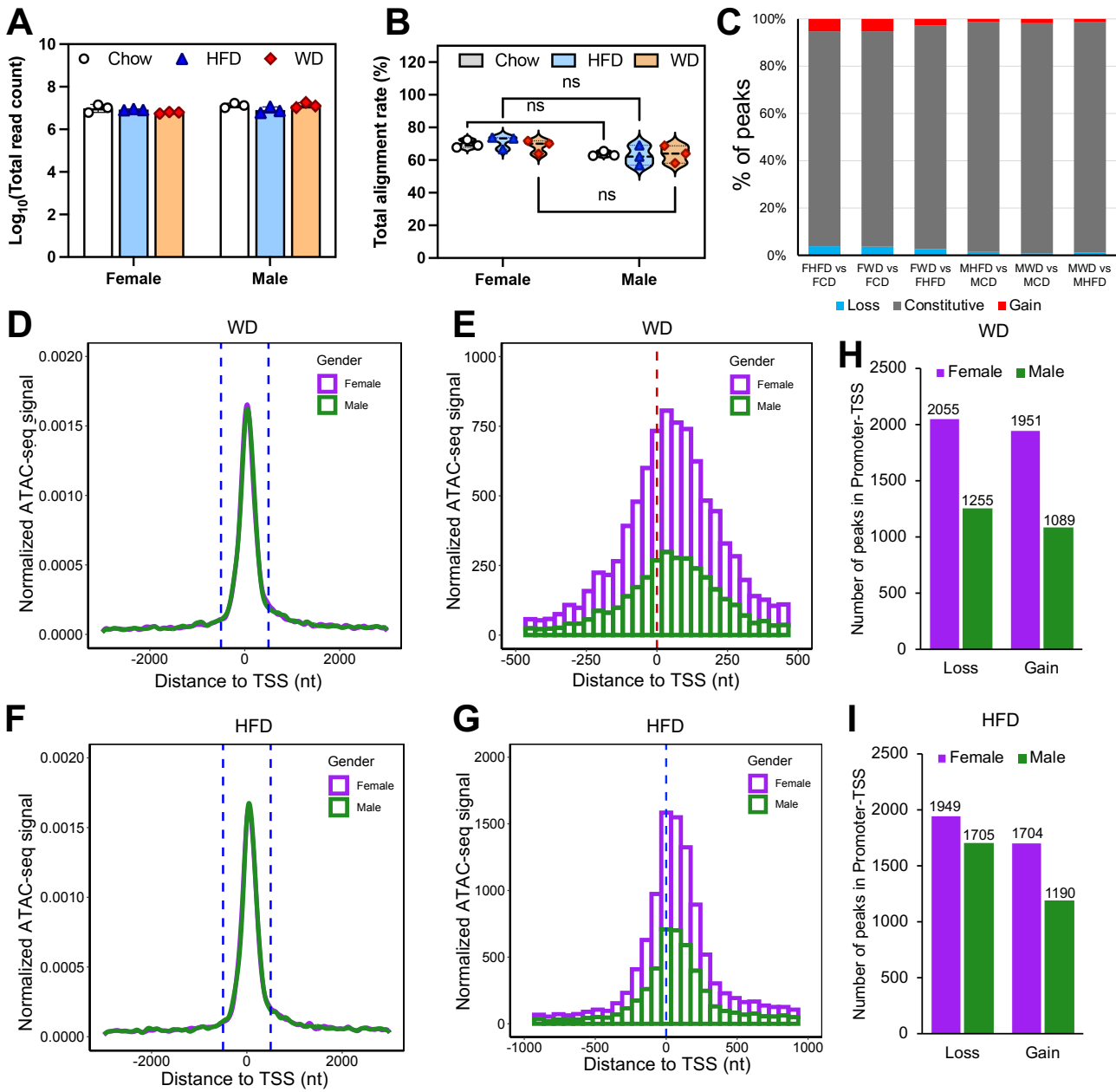

Supplement: S1 Fig — (A) Total read count of each ATAC-seq sample. Values are shown as log10(total read count). (B) Total alignment rate from each sample, calculated with bowtie2 using mm9 as the reference genome. (C) Proportion of total peaks that showed gain, loss, or no change in chromatin accessibility in male (M) and female (F) mice fed a WD or HFD relative mice fed a CD. Gain or loss of accessibility was defined as a fold change (FC) >2 (gain) and P < 0.05, or FC < 0.5 and P < 0.05 (loss). (D and E) ATAC-seq signal intensity of peaks in mice fed a WD relative to that in mice fed a CD. (F and G) ATAC-seq signal intensity of peaks in mice fed a HFD relative to that in mice fed a CD. (H) Number of peaks in the promoter–TSS region that differed in male and female mice, according to HOMER annotation. (I) Number of peaks in the promoter–TSS region that differed in male and female mice, according to HOMER annotation. (PDF) [file pbio.3003601.s008.pdf]

S2 Fig

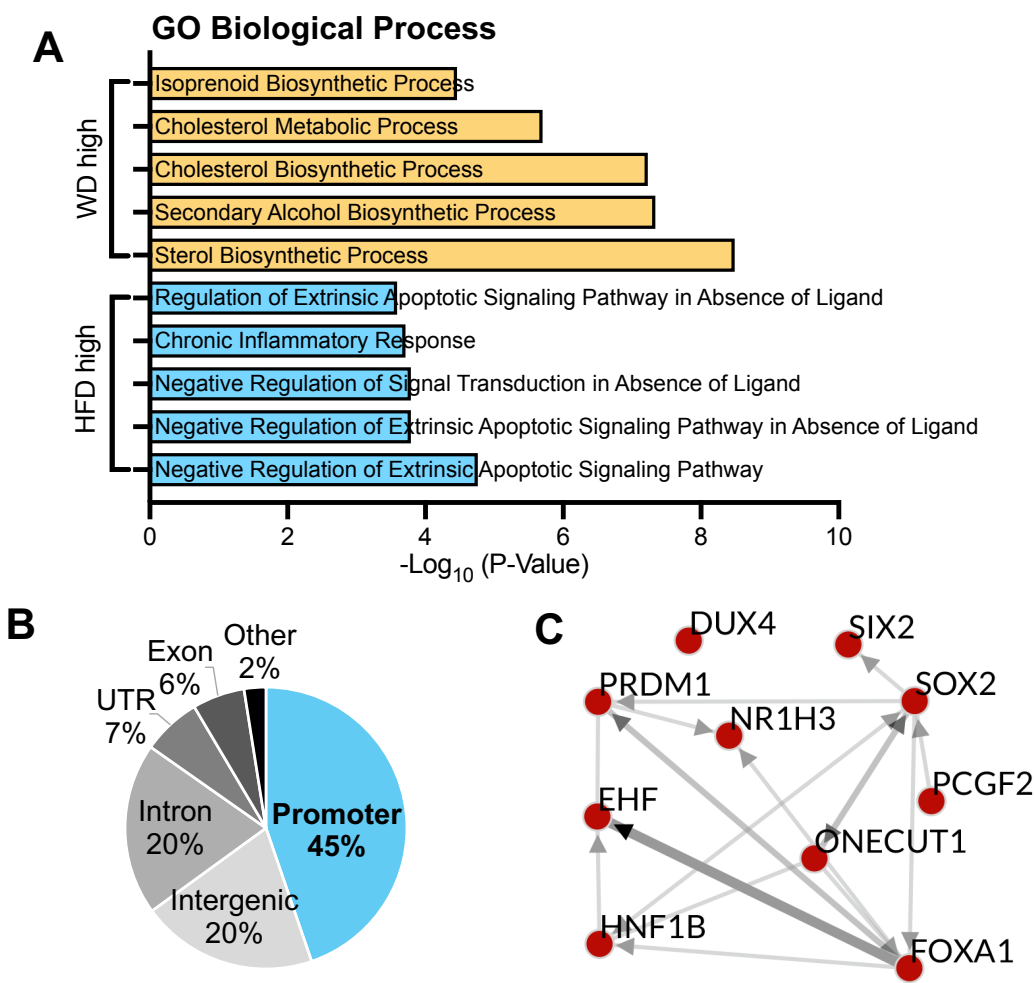

Supplement: S2 Fig — (A) GO annotation (Biological Process) was done using the DEGs from Fig 2F. (B) Distribution of genomic locations for selected peaks in Fig 3B. (C) CHEA3 motif prediction using top 300 global peaks (WD versus CD) in male mice (P < 0.05). (PDF) [file pbio.3003601.s009.pdf]

S3 Fig

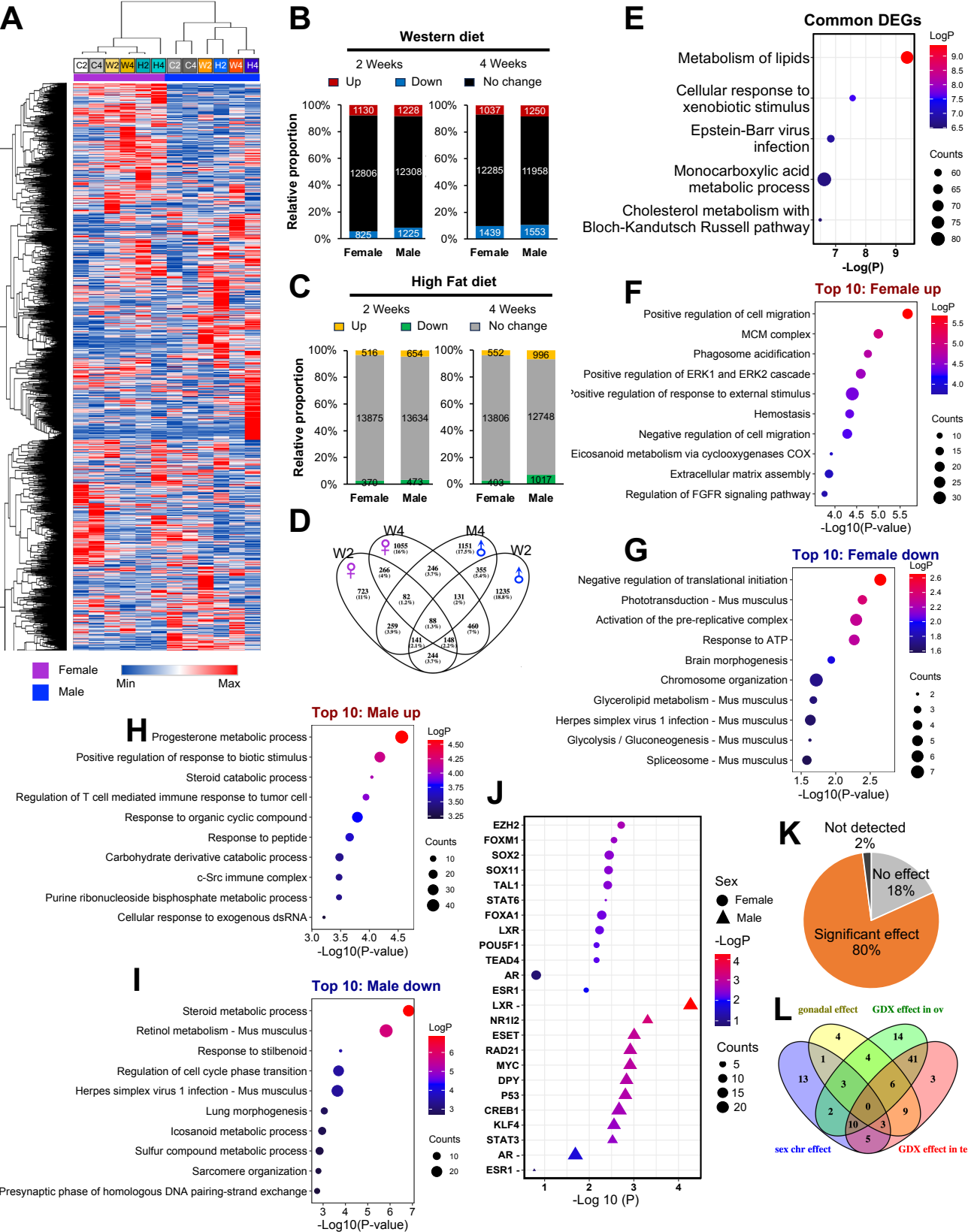

Supplement: S3 Fig — (A) Heatmap of data for all the genes detected by RNA-seq in mouse liver samples. (B) DEGs in male or female mice fed a WD for 2 or 4 weeks. (C) DEGs in male or female mice fed a HFD for 2 or 4 weeks. (D) Venn diagram showing common DEGS in females and males and sex-specific DEGs. (E) Metascape analysis of male and female common DEGs. The top 5 terms are shown. (F–I) Top 10 Metascape terms for female- or male-specific upregulated or downregulated genes. (J) Motifs enrichment prediction of male-biased or female-biased genes in Fig 4F. (K) Genes from Fig 4F matched to DEGs identified by RNA-seq in the FCG mouse model. P < 0.05 was considered significant. (L) Venn diagram of diet-induced sex-difference genes from Fig 4F and DEGs identified by RNA-seq in the FCG mouse model. (PDF) [file pbio.3003601.s010.pdf]

# S4 Fig

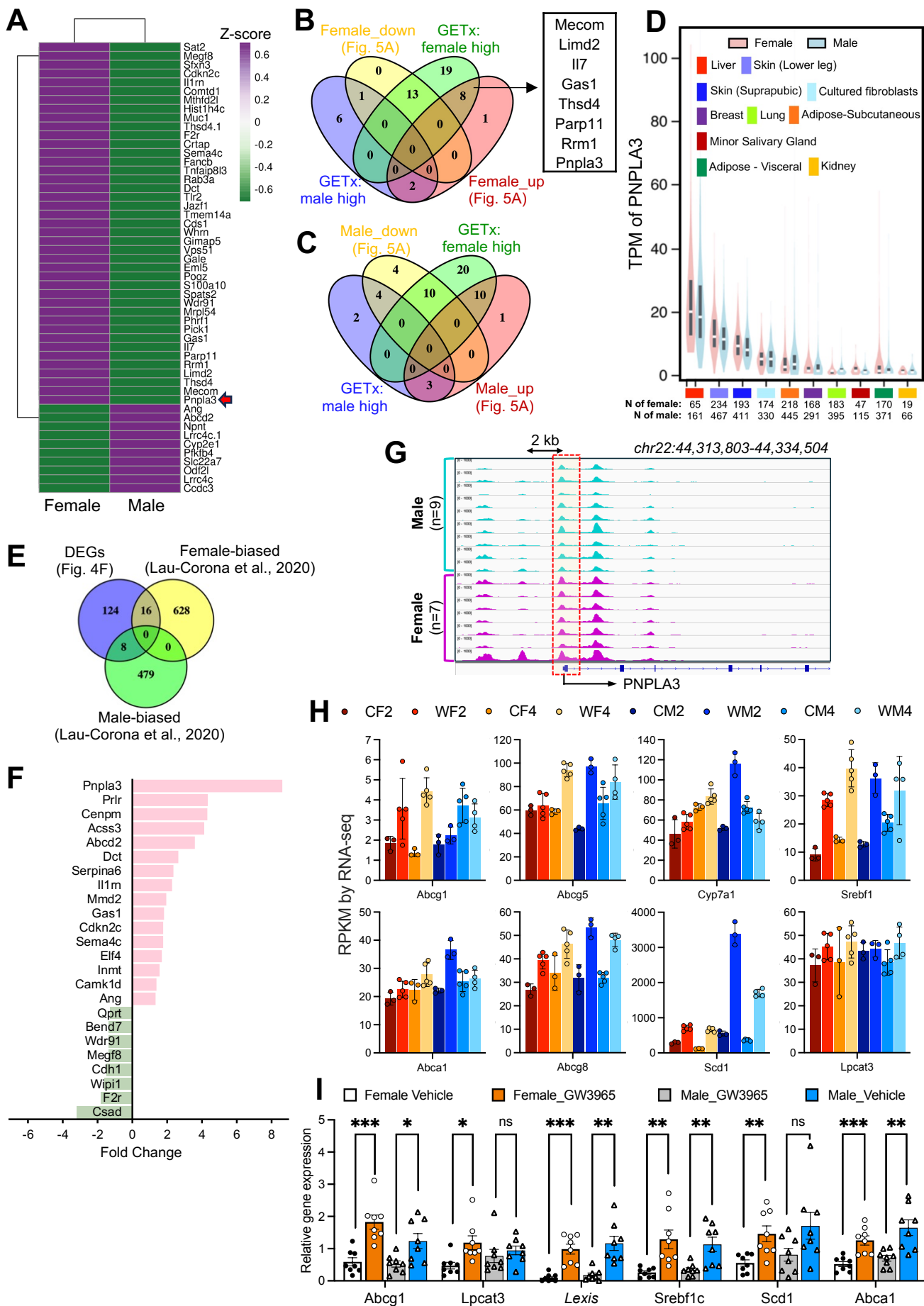

Supplement: S4 Fig — (A) Heatmap of transcripts per million reads (TPM) of genes (listed in Fig 5A) in human liver. TPMs from Adult Genotype Tissue Expression (GTEx) Project. Genes from Fig 5A that had no corresponding human genes or were not express were removed. (B and C) Venn diagram of diet-induced DEGs (Fig 5A) and female-biased (B) and male-biased (C) GTEx genes. (D) Expression of human PNPLA3 in top 10 tissues. Data from GTEx. (E) Venn diagram of DEGs (Fig 4F) and sex-biased hepatic genes. (F) Fold change (male/female relative to controls) of sex-biased DEGs in S4E Fig determined with data from Lau-Corona and colleagues, 2020. (G) Chromatin accessibility by ATAC-seq from human liver samples (GEO accession: GSE164870). All samples from study were included except samples with IDs 767 (GSM5021293), 485 (GSM5021297), 793 (GSM5021300), and 797 (GSM5021301) due to globally reduced peak signals observed in these datasets. (H) LXR target gene expression based on RNA-seq. (CF2 and WF2): female mice fed a CD or WD for 2 weeks, (CF4 and WF4), females fed a CD or WD for 4 weeks, (CM2 and CM4) males fed a CD for 2 or 4 weeks, (WM2 and WM4) males fed a WD for 2 or 4 weeks. Number of samples for each group are listed in Table 1. (I) Expression of LXR target genes detected by qPCR from liver. n = 8 per group. *P < 0.05, **P < 0.01, ***P < 0.001 by unpaired t test. (ns) not significant. The data underlying this Figure can be found in S1 Data. (PDF) [file pbio.3003601.s011.pdf]

S5 Fig

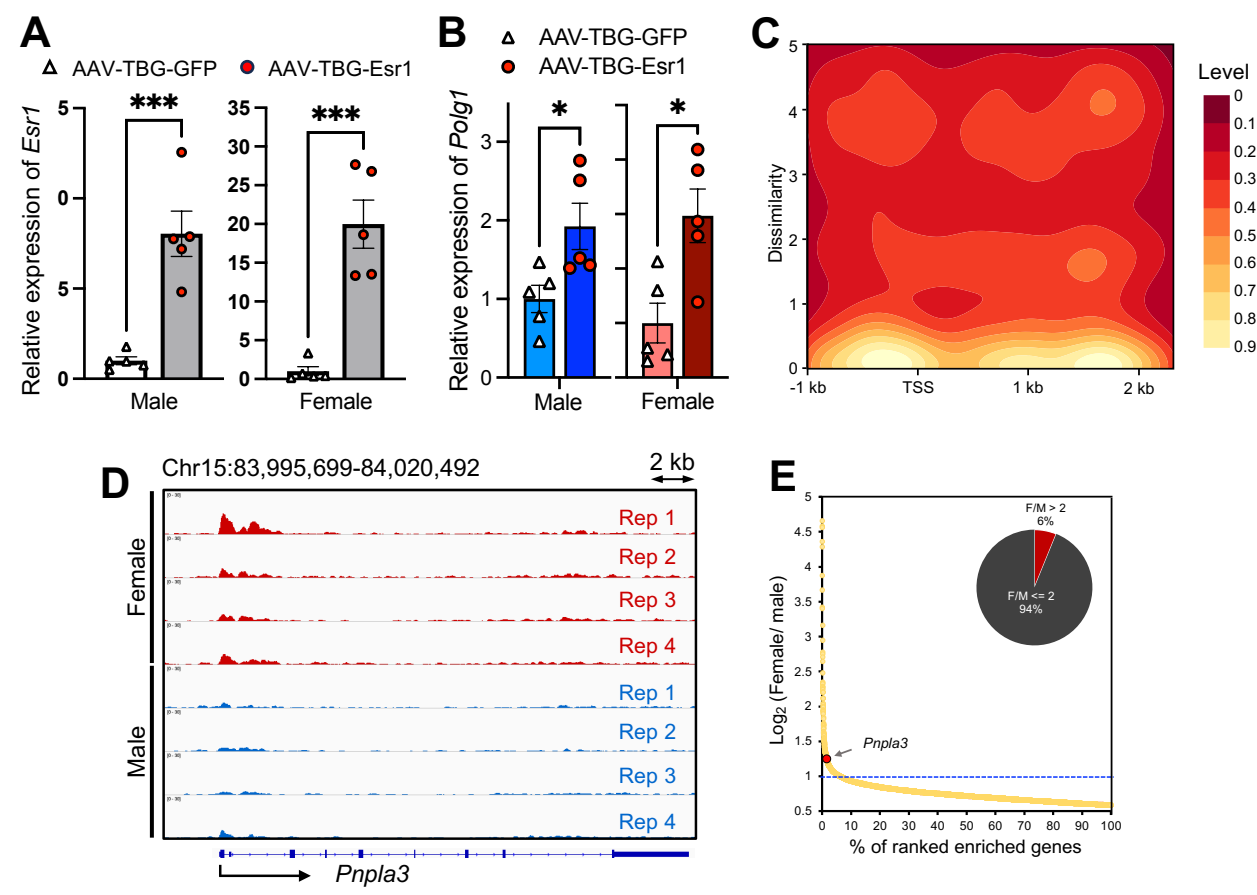

Supplement: S5 Fig — (A) Hepatic expression of Esr1 of mice from Fig 5F, determined by qPCR. n = 5. ***: P < 0.001 by unpaired t test. (B) Hepatic expression of Polg1 of mice from Fig 5F, determined by qPCR. n = 5. *: P < 0.05 by unpaired t test. (C) PROMO-predicted TF binding sites around the TSS of Pnpla3, as indicated in the red box in Fig 5E. “Level” is the density of binding sites of TFs; a higher level indicates greater TF binding. (D) H3K27ac ChIP-seq peaks from public datasets with the following GEO ID numbers: SRR6756579 (female Rep 1), SRR6756580 (female Rep 2), SRR6756581 (female Rep 3), SRR6756582 (female Rep 4), SRR6756571 (male Rep 1), SRR6756572 (male Rep2), SRR6756573 (male Rep3), and SRR6756574 (male Rep 4). (E) Fold change calculated based on ChIP-seq enrichment in acetylation of lysine 27 of the histone H3 protein (H3K27ac) in male or female liver (Lau-Corona and colleagues, 2020). SeqMonk was used for gene annotation (mRNA). The data underlying this Figure can be found in S1 Data. (PDF) [file pbio.3003601.s012.pdf]

S6 Fig

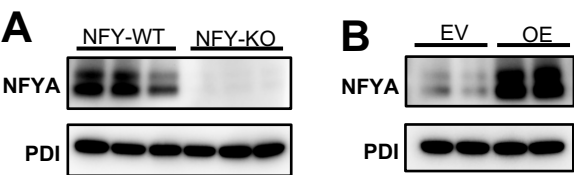

Supplement: S6 Fig — (A) NFYA protein levels detected by immunoblotting. Protein was isolated 48 h post AAV infection (Materials and methods for details). Twenty micrograms of total protein were loaded per lane. (B) NFYA overexpression efficiency detected by immunoblotting. Primary hepatocytes from WT mice were transfected with empty vector (EV) or NFYA overexpression plasmid (OE). Protein was isolated 36 h post-transfection, with 15 µg total protein loaded per lane. These cells were further used for ChIP in Fig 6E. (PDF) [file pbio.3003601.s013.pdf]
